# Causes of individual differences in adolescent optimism: a study in Dutch twins and their siblings

**DOI:** 10.1007/s00787-015-0680-x

**Published:** 2015-02-01

**Authors:** Rezan Nehir Mavioğlu, Dorret I. Boomsma, Meike Bartels

**Affiliations:** 1 Department of Molecular Biology and Genetics, Boğaziçi University, Istanbul, Turkey; 2 Department of Biological Psychology, Netherlands Twin Register, VU University Amsterdam, Van der Boechorststraat 1, 1081 BT Amsterdam, The Netherlands; 3 EMGO+ Institute for Health and Care Research, VU University Medical Centre, Amsterdam, The Netherlands; 4 Neuroscience Campus Amsterdam, Amsterdam, The Netherlands

**Keywords:** Optimism, Pessimism, Twin-sibling design, Adolescence, Non-shared environment

## Abstract

The aim of this study was to investigate the degree to which genetic and environmental influences affect variation in adolescent optimism. Optimism (3 items and 6 items approach) and pessimism were assessed by the Life Orientation Test-Revised (LOT-R) in 5,187 adolescent twins and 999 of their non-twin siblings from the Netherlands Twin Register (NTR). Males reported significantly higher optimism scores than females, while females score higher on pessimism. Genetic structural equation modeling revealed that about one-third of the variance in optimism and pessimism was due to additive genetic effects, with the remaining variance being explained by non-shared environmental effects. A bivariate correlated factor model revealed two dimensions with a genetic correlation of −.57 (CI −.67, −.47), while the non-shared environmental correlation was estimated to be −.21 (CI −.25, −.16). Neither an effect of shared environment, non-additive genetic influences, nor quantitative sex differences was found for both dimensions. This result indicates that individual differences in adolescent optimism are mainly accounted for by non-shared environmental factors. These environmental factors do not contribute to the similarity of family members, but to differences between them. Familial resemblance in optimism and pessimism assessed in adolescents is fully accounted for by genetic overlap between family members.

## Introduction

Increasing attention and effort has been directed to positive aspects of human behavior and functioning. Optimism research has been a significant part in positive psychology [1, 2]. The trait of optimism is generally defined as expecting positive outcomes in any life event or situation. Test–retest correlations of optimism are high for long periods of time [3], indicating that it is a stable trait. Optimism has been associated with many mental and physical health conditions, and multiple dimensions of personality. Optimism is related to lower depression levels [4, 5], less academic stress [6], coping [7], and higher health-related quality of life [8, 9]. It is linked to other phenotypes like happiness [10], self esteem [11], subjective wellbeing [12], hope [13], and life satisfaction [14]. Optimism also is a positive resource for social relationships [2]. Optimism predicts positive physical health outcomes [15]. It is associated with lower levels of pain [16], greater serum antioxidants [17], and healthy lipid profiles [18].

Seligman proposed that optimism can be learned by experience [10], while twin studies suggest that optimism is a partly heritable trait, although heritability estimates tend to be low to moderate. These two observations are not necessarily exclusive, since the variance in most complex traits results from a combination of genetic influences, environmental influences, and their interplay [19].

The oldest twin-adoption study of individual differences in optimism and pessimism, as assessed with the Life Orientation Scale [20], found that about 25 % of the variance was explained by genetic differences between people [21]. Later, using the Attributional Style Questionnaire (ASQ; [22]), Schulman and Keith [23] found further evidence for genetic influences on optimism. In a small sample of 115 monozygotic and 27 dizygotic twin pairs, they observed a monozygotic twin correlation of 0.48, but a dizygotic twin correlation of 0. More recent studies have used the Life Orientation Test-Revised (LOT-R; [24]), reporting estimates of about 20–36 % for additive genetic influences on optimism, with the remaining variance accounted for by non-shared environmental factors. Almost all studies on the mental and physical factors associated with optimism and on the causes of individual differences in optimism have been based on adult samples. Plomin et al. [21] reported a heritability estimate of 25 % in a sample with an age range of 26–91. Mosing et al. [25, 26] found heritability estimates of 36 and 33 % in their 2009 and 2010 studies, respectively, using twins aged 50–94 and the sample from Plomin et al., in their 2010 follow-up study. Caprara et al. [27] and Alessandri et al. [28] reported heritability estimates of 28 and 20 %, respectively, in samples of young adults. Much less is known about the role of optimism in adolescent development, and it is unclear if the genetic architecture of optimism during adolescence is similar to the genetic architecture during adulthood.

In addition, it has been suggested that the revised version of the Life Orientation Test (LOT-R) suffers from dimensionality problems, which is the focus of an ongoing debate. Factor analyses have revealed two correlating components, but it is not clear what these components actually measure nor how researchers should calculate an optimism score using the LOT-R. Generally, there are four views on this problem: (1) there are two distinct components which represent optimism and pessimism, and the scale should be treated as bi-dimensional (i.e., separate scores for optimism and pessimism) [29–31], (2) two dimensions represent optimism and social desirability [32] or positive method effect [28] and the optimism score should be calculated that way, (3) two factors symbolize optimism and pessimism but all three scores should be used (total score of LOT-R, summation of positive items for optimism, summation of negative items for pessimism) [33], and (4) optimism and pessimism are opposite poles of a continuous trait, and one should use the uni-dimensional model and calculate total LOT-R score for optimism; but if necessary, bi-dimensional model can be used [24, 34]. Related to this view, in their correlational study, Segerstrom et al. [35] found that in terms of personality traits, scores on optimism and pessimism components of LOT-R did not differ except a little difference in cheerfulness. Their suggestion was to treat the scale as uni-dimensional and to calculate a total optimism score from LOT-R. To gain more insight into the dimensionality of the scale, we analyzed both the full scale (Optimism_6 items) and the two subscales (Optimism_3 items and Pessimism_3 items). In addition, we analyzed the optimism and pessimism subscales simultaneously in a correlated factor model to test for overlap and differences in genetic and environmental influences. In the present study, we analyzed the LOT-R in a large sample of 13–16-year-old twins and their non-twin siblings from the Netherlands Twin Register (NTR). We aim to disentangle genetic and environmental influences on optimism, pessimism, and their overlap.

## Materials and methods

### Participants

Participants were registered at birth with the Netherlands Twin Register (NTR; [36]). For this study, data from the surveys collected in 13–16-year-old twins and their non-twin siblings were used. The sample consisted of 5,187 adolescent twins (2,142 twin pairs and 903 individuals whose co-twin did not participate, 42.3 % male) and 999 non-twin siblings (41.2 % male), resulting in a total sample size of 6,186 individuals (42.09 % male). The sample included 756 MZM twins (319 complete pairs), 691 DZM twins (278 complete pairs), 1,116 MZF twins (495 complete pairs), 939 DZF twins (398 complete pairs), and 1,685 twins of opposite sex (652 complete pairs) (for an overview see Table 1). The twins’ mean age was 14.75 (SD = 0.71, range 13–16.6) and the siblings’ mean age was 17.44 (SD = 0.71, range 12–25.7). Zygosity of same-sex twin pairs was determined by discriminant analysis, using longitudinally assessed questionnaire items from the previously collected parental and self-reports. Agreement between zygosity assignment based on questionnaire information and zygosity determined by DNA markers was around 93 % [37]. In a subsample (20 %), zygosity was determined by DNA or blood group analysis. This study was reviewed and approved by the Medical Ethics Review Committee of the VU University Medical Center Amsterdam (2003/182).

**Table 1:** Sample size and descriptives for the full sample and for males and females separately

Family constitution	Number of families	Number of individuals
Twin pair with a brother and sister	42	168
Twin pair, no sibling	1,483	2,966
Oldest of the twin pair	302	302
Youngest of the twin pair	446	446
Twin pair with brother	269	807
Twin pair with sister	348	1,044
Oldest of the twin with brother	23	46
Oldest of the twin with sister	38	76
Oldest of the twin with brother and sister	3	9
Youngest of the twin with brother	28	56
Youngest of the twin with sister	60	120
Youngest of the twin with brother and sister	3	9
Brother only	38	38
Sister only	87	87
Brother and sister (no twin)	6	12
Total	3,176	6,186

Descriptives	*N*	Mean	SD	Skw	SE Skw	Kur	SE Kur
Full sample							
Optimism_6 items	6,186	20.84	3.09	−.194	.031	.516	.062
Optimism_3 items	6,186	10.38	1.78	−.258	.031	.588	.062
Pessimism_3 items	6,186	7.55	2.02	.291	.031	.069	.062
Males							
Optimism_6 items	2,604	21.21	3.03	−.202	.048	.370	.096
Optimism_3 items	2,604	10.68	1.74	−.272	.048	.834	.096
Pessimism_3 items	2,604	7.47	2.02	.327	.048	.099	.096
Females							
Optimism_6 items	3,582	20.56	3.11	−.180	.041	.629	.082
Optimism_3 items	3,582	10.16	1.78	−.244	.041	.473	.082
Pessimism_3 items	3,582	7.60	2.02	.265	.041	.056	.082

Parents of twins and siblings 16 years of age and younger were contacted to ask for permission to send their children a self-report survey and to register non-twin siblings of the twins. Upon parental consent, questionnaires were sent to the twins and their siblings. Initially, the survey was presented in a paper and pencil version. In 2009, data collection continued with an online version of the questionnaire and the paper and pencil version was used as a reminder. Response rate was 47 %. Non-response analyses showed that familial and parental characteristics such as socioeconomic status, smoking, and alcohol consumption during pregnancy, religious beliefs, and parental education were not different for the families of responding versus non-responding twins. At the ages of 7 and 12, non-respondent twins had slightly higher externalizing behavior scores than respondents (8.6 vs. 7.4 at age 7; 6.5 vs. 5.0 at age 12) [38].

### Measures

The Life Orientation Test (LOT-R; [24]) is a measure of optimism which is included in the Dutch Health Behavior Questionnaire (DHBQ), a self-report instrument containing a broad range of measures on health, lifestyle, and behavior [39]. The LOT-R is a ten-item scale which has three positive and three negative 5-point Likert-type scale items (1 = strongly disagree, 5 = strongly agree) and four filler items. Two example items are “In uncertain times, I usually expect the best” and “I rarely count on good things happening to me.” Internal consistency of the scale was acceptable, with a Cronbach’s Alpha of 0.65. Exploratory factor analysis revealed two correlated factors (*r* = −.30), explaining 55.54 % of the variance. Negative and positive items were clustered with different factors. In the current study we calculated optimism based on the three positive items (Optimism_3 items), Pessimism based on the 3 negative items (Pessimism_3 items) and a total optimism score where the negative items were reverse scored and added to positive items (Optimism_6 items). For the latter, higher total scores indicate higher levels of optimism.

### Descriptive statistics

A saturated model in OpenMx [40] was used to test equality of means and variances in twins and siblings, to test for sex differences in mean levels of optimism scales and pessimism, and to estimate twin and twin-sibling correlations (with 95 % confidence intervals).

### Genetic modeling

Monozygotic twins (MZ) share (nearly) all their genetic information while dizygotic twins (DZ) and twin-sibling pairs share on average the half of their segregating genes. The different degree of genetic relatedness between MZ twin pairs, compared to DZ twin pairs and sibling pairs provides the opportunity to disentangle the variance in optimism and pessimism into genetic and environmental components. Additive genetic variance (A) stands for the additive effects of alleles at each genetic locus. Dominant/non-additive genetic variance (D) includes the non-additive effects or interactions of alleles at genetic loci. Shared environmental variance (C) stands for the effects which result from sharing the same familial environment. Non-shared environmental variance (E) results from environmental experiences, which are unique to each individual. Note that in the classical twin design or in the twin-sibling design the non-additive genetic influences (D) and shared environmental influences (C) are confounded. Non-additive genetic effects increase differences between MZ and DZ covariances, whereas shared environmental variance decreases such differences. Twin-sibling correlations indicate which of the two components is more likely to be present. When DZ-sib correlations are less than half of MZ correlations, dominance is indicated, because D correlates perfectly for MZ but only 25 % for DZ twin- and sib pairs. In contrast, shared environmental influences make DZ-sib correlations greater than half of MZ correlations. Based on the observed twin-sibling correlations, no clear distinction between an ACE and an ADE model could be made. The process of fitting genetic models started with an ACE model with quantitative sex differences, which allowed for differences between boys and girls in the magnitudes of the genetic, and shared and unique environmental estimates. Quantitative sex differences were tested by constraining A, C, and E path loadings to be equal between boys and girls. Next, ADE models with and without sex differences were fit to the data. Finally, significance of shared environmental influences and non-additive genetic influences were tested by constraining either the C component or the D component to zero. After establishing the best-fitting model, 95 % confidence intervals were estimated for all parameters.

To gain more insight into the uni- or bi-dimensionality of optimism and pessimism, we fitted a correlated factor model to the data. In this model the variance in optimism and pessimism is disentangled into genetic and environmental components and these components are allowed to be correlated, providing insight into the overlap of genetic and environmental influences. Higher genetic and environmental correlations provide evidence for overlapping sets of underlying genes and environmental influences and are more in line with a uni-dimensional model. Lower genetic and environmental correlations indicate biologically and environmentally distinct constructs indicative of a bi-dimensional model.

The fit of the different models (both for the saturated as well as the genetic models) was compared by means of the log-likelihood ratio test (LRT). The difference in minus two times the log-likelihood (−2LL) between two nested models has a *χ* ^2^ distribution with the degrees of freedom (*df*) equaling the difference in *df* between the two models. If a *p* value higher than 0.05 was obtained from the *χ* ^2^ test, the fit of the constrained model was considered not significantly worse than the fit of the more complex model. In this case, the constrained model was kept as the most parsimonious and best-fitting model. Since the ACE model and the ADE model are not nested, the test for sex differences was based on the nested ACE or ADE model and the AE model was compared to the ACE model.

## Results

### Descriptive statistics

Saturated model fitting indicated no birth order or zygosity differences in mean levels of optimism and pessimism. Males reported significantly higher mean optimism scores than females both in full scale (Optimism_6; 21.21 vs 20.56; *χ* ^2^(1)  = 63.49, *p* < .01) and optimism subscale (Optimism_3; 10.68 vs 10.16; *χ* ^2^(1)  = 133.57, *p* < .01). Females reported significantly higher mean pessimism scores than males (Pessimism_3; 7.60 vs 7.47; *χ* ^2^ = 5.81, *p* < .05).

### Twin and twin-sibling correlations

Estimated twin-sibling correlations with 95 % confidence intervals are presented in Table 2. Monozygotic twin correlations were higher than dizygotic twin correlations for all scales for both males and females, suggesting genetic influence. No difference in correlations was found between same-sex dizygotic twins and same-sex siblings. Estimated correlations between brothers and sisters were similar to dizygotic opposite-sex twin correlations. No qualitative sex differences are to be expected since the opposite-sex twin correlations were in line with the same-sex twin correlations.

**Table 2:** Estimated twin-sibling correlations for optimism scores with 95 % confidence intervals

Zygosity	Optimism 6 items	Optimism 3 items	Pessimism 3 items
MZM	.32 (.22, .41)	.25 (.15, .35)	.29 (.19, .38)
DZM/MM	.13 (.05, .21)	.06 (−.02, .13)	.13 (.05, .21)
MZF	.42 (.34, .48)	.31 (.23, .39)	.38 (.31, .45)
DZF/FF	.22 (.15, .28)	.21 (.14, .27)	.17 (.10, .23)
DOSmf/MF	.19 (.14, .24)	.15 (.09, .20)	.17 (.12, .23)

### Genetic model fitting

Univariate model fitting results for Optimism_6 items, Optimism_3 items, and Pessimism_3 items are presented in Table 3. The ACE model with sex differences gave a good fit in comparison to the saturated model and provided initial insight into the causes of differences in optimism and pessimism scores. Constraining the path coefficients to be equal for males and females gave no significant deterioration of the fit of the model for Optimism_6 items and Pessimism_3 items (all *p* values ≥.05). For Optimism_3 it was not clear if sex differences were important or not since the *p* value was .05. The standardized variance components, however, were rather similar (A_m_: .21, A_f_; .23; C_m_: .01, C_f_; .08; E_m_: .78, E_f_; .68) and model fitting was continued with a model without sex differences for all three phenotypes. The ADE model provided a similar picture, with no significant sex differences for any of the phenotypes. Dropping the shared environmental component or the non-additive genetic component did not result in a significant change in model fit (all *p* values ≥.05). An AE model without sex difference was chosen as the best-fitting model for all scales.

**Table 3:** Genetic model fitting results

		Model	−2LL	*df*	c.t.m.	*χ* ^2^	Δ*df*	*p*
Optimism_6 items	1	Saturated	30,535.02	6,040				
	2	ACE with sex differences	30,535.46	6,041	1	.44	1	.51
	3	ADE with sex differences	30,535.50	6,041	1	.48	1	.49
	4	ACE no sex differences	30,541.17	6,044	2	5.71	3	.13
	5	ADE no sex differences	30,541.14	6,044	3	5.65	3	.13
	6	AE no sex differences	30,541.17	6,045	4	.0	1	1.0
	7	AE no sex differences	30,541.17	6,045	5	.03	1	1.0
Optimism_3 items	1	Saturated	23,889.97	6,040				
	2	ACE with sex differences	23,892.8	6,041	1	2.83	1	.09
	3	ADE with sex differences	23,893.99	6,041	1	4.02	1	.05
	4	ACE no sex differences	23,900.47	6,044	2	7.67	3	.05
	5	ADE no sex differences	23,900.48	6,044	3	6.49	3	.09
	6	AE no sex differences	23,900.48	6,045	4	.01	1	1.0
	7	AE no sex differences	23,900.48	6,045	5	.0	1	1.0
Pessimism_3 items	1	Saturated	25,471.47	6,040				
	2	ACE sex differences	25,472.14	6,041	1	.67	1	.41
	3	ADE with sex differences	25,472.01	6,041	1	.54	1	.46
	4	ACE no sex differences	25,474.97	6,044	2	2.83	3	.42
	5	ADE no sex differences	25,474.84	6,044	3	2.83	3	.42
	6	AE no sex differences	25,474.97	6,045	4	.0	1	1.0
	7	AE no sex differences	25,474.97	6,045	5	.13	1	1.0

*c.t.m* compared to model

In Table 4, standardized estimates and 95 % confidence intervals of genetic and environmental variance components for optimism and pessimism are presented for the ACE, ADE, and AE models. According to the best-fitting (AE) model for the full scale, the additive genetic influence on optimism was around 38 %, whereas the influence of non-shared environment was 62 % for both males and females. For the optimism subscale, the additive genetic influence was 29 %, and the influence of non-shared environment was 71 %. The additive genetic influence on pessimism was 34 %. The effect of non-shared environment on pessimism was 66 %.

**Table 4:** Standardized estimates with 95 % confidence intervals

	ACE model_ univariate			ADE model_univariate			AE model_univariate		AE model_bivariate	
	A	C	E	A	D	E	A	E	A	E
Optimism_6 items	.38 (.25, .42)	.00 (.00, .09)	.62 (.58, .68)	.37 (.21, .42)	.01 (.00, .20)	.62 (.56, .67)	.38 (.33, .42)	.62 (.57, .67)		
Optimism_3 items	.28 (.14, .34)	.01 (.00, .10)	.71 (.66, .78)	.29 (.13,.34)	.00 (.00, .18)	.71 (.65, .76)	.29 (.24, .34)	.71 (.66, .76)	.30 (.25, .35)	.70 (.65, .75)
Pessimism_3 items	.34 (.22, .39)	.00 (.00, .8)	.66 (.61, .72)	.31 (.14, .38)	.04 (.00, .22)	.66 (.60, .71)	.34 (.29, .39)	.66 (.61, .71)	.34 (.29, .39)	.66 (.61, .71)

Based on the results of the univariate analyses, a bivariate AE correlated factor model without sex differences was fit to the data. The C and D components were not included due to the absence of influences both for optimism as well as for pessimism and estimates were constrained to be similar for boys and girls due to the absence of evidence for quantitative sex differences in the univariate analyses. As expected the heritability estimates and the estimate for the influences of non-shared environment are identical to the results of the univariate analyses (see Table 4). Furthermore, the model showed significant overlap in genetic and environmental influences. The genetic correlation was estimated at −.57 (CI −.67, −.47), while the non-shared environmental correlation was estimated to be −.21 (CI −.25, −.16).

## Discussion

The objective of this study was to disentangle the causes of individual differences in optimism (measured with 3 items or measures with 6 items) and pessimism (3 items) and to gain insight into uni- versus bi-dimensionality of the LOT-R in an adolescent sample. Additive genetic influences accounted for about one-third of the variance in optimism. Using an optimism score based on 6 items resulted in a slightly higher heritability estimate than when a measure with 3 items was used, although the 95 % confidence intervals were overlapping. Given the similar reliability of the 6-item and the 3-item scales of the LOT-R in our study (*α* = .65 and .54 for the 6-item and 3-item optimism scales), and also in other studies, e.g., [30, 31], this difference is not expected to be due to a difference in measurement error. The heritability of pessimism is in line with the heritability of optimism, and additive genetic factors also account for about one-third of its variance. The remaining variance in both optimism measures and in pessimism is accounted for by non-shared environmental influences, including measurement error.

The absence of significant influences of shared environmental or non-additive genetic influences is often due to a lack of power to detect this source of variance. However, our study included non-twin siblings and thereby increased the power to detect small shared environmental or non-additive genetic effects. Given our sample size, shared environmental effect or non-additive genetic effects should have been detected if present [41].

The significant and substantial effect of non-shared environment supports the view of Seligman that optimism can be learned by experience, which supports including optimism in prevention and intervention strategies.

Our results in an adolescent sample are in line with previous studies that used samples of (young) adults or elderly participants in both optimism and pessimism constructs [21, 25, 27, 28, 42]. The heritability of some traits and behaviors is subject to change over the lifetime because of the decaying effect of shared environment [43–45], and because of some life events that may or may not lead to gene–environment interactions. Comparison of the previous studies on optimism with our findings reveals that age seems to have no effect on the genetic architecture of optimism. Therefore, optimism seems to be a stable trait over lifetime.

The finding of a heritability estimate of about 38 % for the full scale (6 items) and 29 % for the Optimism_3 items subscale is in accordance with estimates for other positive psychology measures, such as subjective wellbeing and its components, happiness, satisfaction with life, and quality of life [46, 47]. Some of these studies, though, show evidence for non-additive genetic influences, which seem to have been of no significance to optimism in this large extended twin-family sample.

The results of the bivariate analyses add significant knowledge to the field by revealing that optimism and pessimism cannot be considered to be uni-dimensional. The genetic and environmental correlations indicate that the Life Orientation Test (LOT-R) captures two correlated components.

In conclusion, the LOT-R should be considered a bi-dimensional scale with two correlated constructs. Whether the full LOT-R or the subscale of 3 optimism items is used, about one-third of the variance is accounted for by additive genetic differences between individuals, while the remaining variance is accounted for by non-shared environmental influences.
